# Forward first: Joystick interactions of toddlers during digital play

**DOI:** 10.1371/journal.pone.0316097

**Published:** 2024-12-19

**Authors:** Kimberly A. Ingraham, Heather A. Feldner, Katherine M. Steele

**Affiliations:** 1 Department of Electrical & Computer Engineering, University of Washington, Seattle, WA, United States of America; 2 Division of Physical Therapy, Department of Rehabilitation Medicine, University of Washington, Seattle, WA, United States of America; 3 Department of Mechanical Engineering, University of Washington, Seattle, WA, United States of America; Mehran University of Engineering and Technology, PAKISTAN

## Abstract

Developmentally appropriate access to technology can support toddlers in learning and play. While touch screens are a popular interaction modality for children under the age of three, they may not be appropriate for all children or all tasks. We know comparatively little about how toddlers interact with joystick-based technology, and more fundamental research is required to understand joystick interactions at different ages and developmental stages. We quantified how 36 nondisabled toddlers used a joystick to play a cause-and-effect game on a computer. Children demonstrated a strong preference for moving the joystick forward first, regardless of the target direction. On average, the oldest children navigated the joystick to the target 5 seconds faster than the youngest children, and were nearly twice as efficient in their joystick path. These findings inform the design of assistive algorithms for joystick-enabled computer play and developmentally appropriate technologies for toddlers.

## Introduction

Toddlers learn by interacting with and exploring the world around them. For children born today, technology is an inextricable part of their early exploration and development. There is growing evidence that adding interactive computer use to a child’s life at a young age (*i.e.,* preschool) can support problem solving [1–4]. However, research is still emerging related to how interaction with digital tools and technology can support early development for toddlers, and how the choice of input device influences their interactions.

Computers and technology are essential tools for supporting the development of toddlers with and without disabilities. By 18-24 months of age, children develop a sense of self, begin to solve problems, grow their ability to use mental abstractions, and understand cause and effect [5–7]. For nondisabled toddlers, small amounts of interactive, age-appropriate computer use may be used to reinforce these developing connections and provide supplemental opportunities for education and play [8]. For toddlers with disabilities, interacting with technology can provide a means of navigating and exploring the world, communicating, participating in play, and experiencing the effects of their own self-initiated actions [9–13]. Augmentative and alternative communication (AAC) devices can be used to support the early stages of communication, and research has consistently demonstrated that these systems can be effectively used by toddlers younger than three years old [11, 14–16]. Technology also affords children with disabilities the opportunity to explore and independently navigate the world through the use of motorized wheelchairs or powered mobility devices [17–19]. Yet, interacting with these technologies in a developmentally appropriate way can be challenging for toddlers, as many are not designed specifically for their use [20, 21].

Since the rise of the personal computer, researchers have been studying how well children can utilize different input devices (*e.g.,* mouse, joystick) for meaningful computer interaction [20–29]. The majority of this work has focused on children over the age of three, with a particular emphasis on school-aged children (4-13 years old) who may use computers for educational activities or for play. Several studies have shown that children’s proficiency using traditional input devices increases with age [21, 22, 24, 25, 27], and that younger children demonstrate higher variability in their performance [21, 25]. Furthermore, some studies have investigated differences in performance based on the child’s gender, but results have been mixed [23, 30]. To date, a small number studies have compared children’s performance between input devices such as mice, joysticks, trackballs or keyboards [21, 23, 24, 30, 31], and only two included children young as three years old [30, 31]. Yet, with the rise in touch screen technology, research on toddlers using interfaces such as joysticks has considerably slowed.

Today, touch screens are by far the most common interface used by toddlers, due to their relative ease of use compared to traditional input devices such as mice [20, 27–29, 32]. It is reported that nearly 40 percent of 2-4 year old children in the United States have used a touch screen smartphone or tablet [33], and many toddlers demonstrate the ability to execute a variety of different gestures (*e.g.,* tap, drag, rotate) using a touch screen [34–37]. However, despite the increasing ubiquity of touch screen interfaces, they may not be the optimal input modality for all young children to interact with technology in all situations. A variety of factors, such as the demands of the task or a child’s age, motor skills, and cognitive development are likely to influence the best choice of input device. Therefore, it is critical that we continue to study how toddlers use physical input devices such as buttons, switches, or joysticks in order to support the future development of age-appropriate technologies for toddlers.

A switch or button is a useful non-touch-screen interface that can be easily used by toddlers to interact with technology. While there has been wide acceptance and deployment of switch-based toys and technology in the community, there have been very few studies quantifying how children interact with switch-based interfaces in a controlled environment. In one study of note, Swinth et al. [38] quantified how nondisabled infants and toddlers (ages 6-17 months), used a single switch to activate a short musical animation on a computer screen. The authors found that children could achieve task success around seven months old, and their performance increased with age. Despite their ease of use, switches are limited by the fact that they often can only be used as a control input for one action at a time, and thus are not appropriate for all tasks.

Joysticks can be used for pointing and can produce proportional control actions for higher dimensional tasks, like interacting with a video game or driving a motorized toy car. In contrast to using a touch screen, joysticks are an indirect interface that introduce an additional level of abstraction between the child’s action and the observed effect. However, at this stage, more fundamental research is needed to understand how toddlers learn to use a joystick and what factors underpin the development of skilled joystick use children at different ages and developmental stages.

Prior research with young children with disabilities provides some of our only knowledge about how children under the age of three use a joystick. Observations from deployments of joystick-controlled pediatric powered wheelchairs demonstrate that children as young as 6-8 months old can interact with a joystick and produce exploratory and goal-directed driving behavior [17–19, 39], and that children are more likely to demonstrate driving proficiency using proportional joystick control compared to non-proportional control options like a switch [40]. However, relatively little research has been conducted to understand how toddlers interact with and learn to use a joystick interface for different tasks, such as engaging with a computer game or digital interface. Understanding how toddlers learn to use and interact with a joystick across multiple tasks is an important step toward designing integrated technologies that equitably support children in exploration and play.

**Fig 1:**
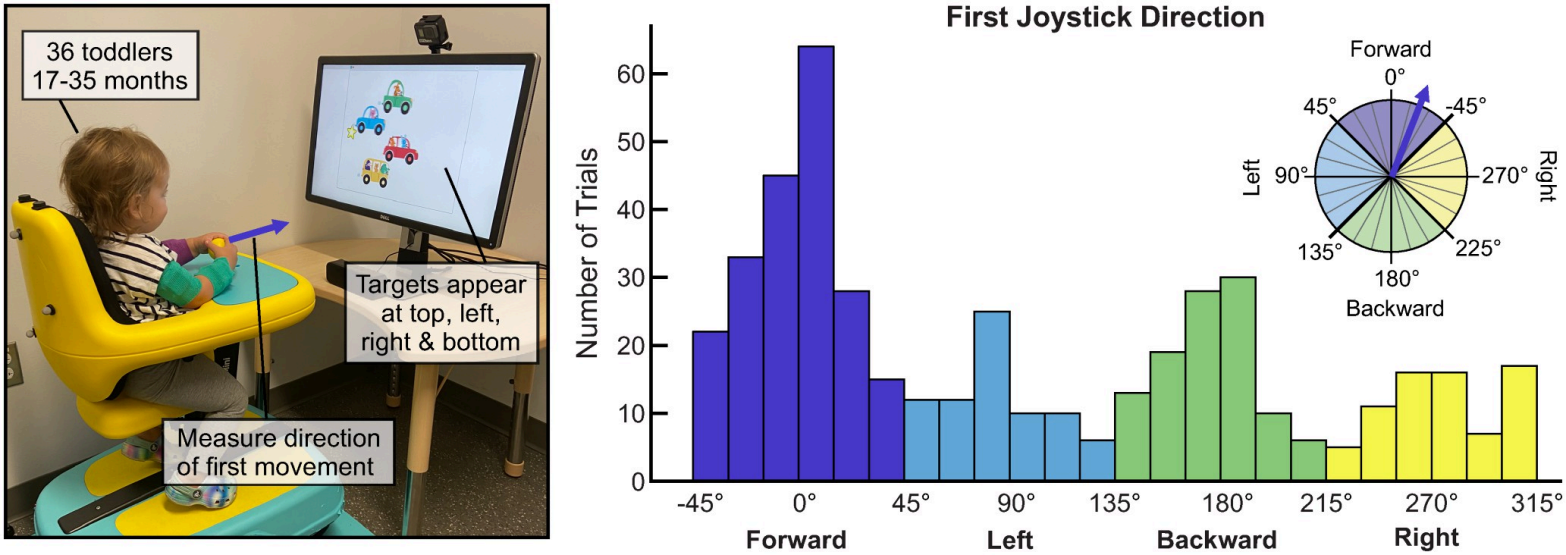
Forward first. When interacting with a joystick to play a directional cause-and-effect game on a computer screen, toddlers demonstrated a strong preference for initiating movement in the forward direction, irrespective of the target on the screen.

The objective of this study is to quantitatively describe how toddlers use a joystick to interact with a computer program, and how their use patterns differ as a function of the child’s age. We tested 36 nondisabled children between the ages of 17 and 35 months playing a simple Scratch-based cause-and-effect game on a computer screen (Fig 1). Children interacted with the program using the joystick of the Permobil Explorer Mini [41], a commercially available mobility technology designed specifically for children 1-3 years old. Our lab has a one-of-a-kind instrumented Explorer Mini that measures joystick position in real time, and we fixed the device to a custom platform so the joystick could be used to control the computer game. This device was an ideal testing environment because of the comfortable, high-chair-like seating system with a tray and a fixed midline-position joystick, all of which are developmentally appropriate for young children ages 1-3. This experience represented the children’s first exposure to a joystick interface. During game play, children saw a target (*i.e.,* monkeys, musical instruments, bubbles, or cars) appear at the top, right, bottom, or left of the screen. Moving the joystick in the direction of the target triggered a short musical animation. Each child was presented with 16 targets (four in each direction), and a successful trial was one in which the child used one or both hands to move the joystick into the target region. We evaluated the effects of age and gender on a variety of quantitative metrics—including time, joystick exploration (*i.e.,* path length), accuracy, and hand use—that described how children used the joystick to play the game. In this paper, we present four key experimental findings:

1. Children demonstrated a strong preference for moving the joystick forward first, regardless of the target direction. Pooled across all ages, participants initiated joystick movement in the forward direction in 45% of trials (Fig 1). This effect was more pronounced amongst the youngest children, who moved forward first in 60% of trials.
2. Task proficiency increased with the child’s age. On average, the oldest children successfully completed 15.6 trials, while the youngest children completed 7.8 trials.
3. Older children successfully moved the joystick into the target region faster than younger children. Our model indicates that 35-month-olds activated the animation 5.1 and 13.6 seconds faster than 17-month-olds, in girls and boys, respectively.
4. Older children moved the joystick less to navigate to the target region compared to younger children, but even the oldest children had difficulty executing efficient paths. On average, the oldest children moved the joystick 7 times farther than the most efficient path to the target, and youngest children moved the joystick 14 times farther.

The primary contribution of this paper is an empirical, quantitative characterization of toddlers’ initial interactions with a joystick interface. The results from this study can directly inform the design of developmentally appropriate joystick-based technologies that can be used to support early access to exploration and play. Furthermore, this study can inform the design of assistive algorithms and design modifications that make joystick-based technology easier or more intuitive for toddlers to use. In the future, our experimental framework may be leveraged to study how toddlers construct cause-and-effect relationships between their physical actions and the observed effects when using an indirect input modality like a joystick. This information could be used to design training programs to help reinforce relationships between the input device and the physical world from a young age.

## Methods

### Participants

We recruited 36 children (20 male, 16 female) without disabilities for this study, aged 17 to 35 months. The recruitment period for this study was open from August 1, 2022 until September 7, 2022. We divided the children into six age groups (A-F, Table 1), where the breaks between age groups were chosen to split the cohort into roughly equal-sized groups. The age range, mean (SD) age, and the number and gender distribution of participants for each group is provided in Table 1. This research involved human participants and the study was approved by the University of Washington Institutional Review Board (STUDY00014879). This study included children ages 12 to 36 months old. Accordingly, the legal parent or guardian gave written informed consent for their child to participate in the study by signing the consent form. Since the children were all below the age of three, assent was not sought from participants, but all efforts were made to explain the study activities to them in an accessible way (for example, using a stuffed animal to demonstrate sitting in the device and using the joystick).

**Table 1:** Study participants.

	Group A	Group B	Group C	Group D	Group E	Group F
Age Range (months)	17-19	20-22	23-26	27-29	30-31	32-35
Mean (SD) Age (months)	18.7 (0.7)	21.6 (0.7)	24.6 (1.1)	28 (0.6)	31.1 (0.6)	34.4 (1.5)
Number of Participants	6	6	6	7	6	5
Gender	3 M, 3 F	2 M, 4 F	3 M, 3 F	6 M, 1 F	3 M, 3 F,	3 M, 2 F

### Game development

We designed a fun and simple cause-and-effect game for children to play using a joystick. We developed the game using Scratch, a block-based, online visual programming language designed primarily for children ages 8-16 years [42]. We combined Scratch’s drag-and-drop code blocks to sense and receive joystick inputs from the user and subsequently control the motion, appearance, and sound of on-screen graphical objects (“sprites”). In our current study, children used the joystick to interact with a pre-constructed program.

**Fig 2:**
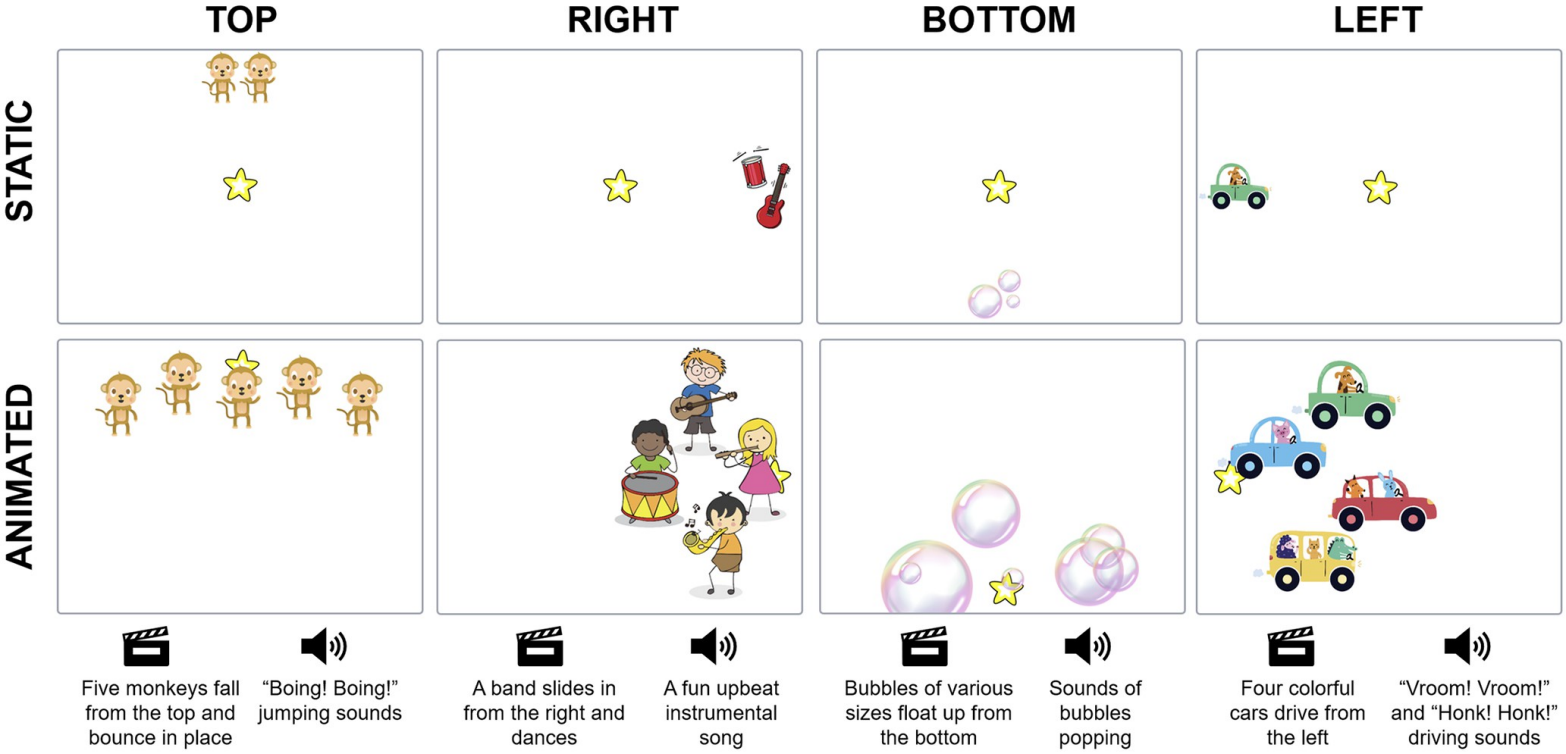
Scratch animations built for this experiment. The top row (static) shows the screen the children saw when a new target appeared. The bottom row (animated) shows still frames of the animations that played when the child successfully moved the joystick into the target region. A description of the animation (indicated by the video marker icon) and the sound (indicated by the speaker icon) is provided for each target direction.

The high-level goal of the game was for children to move the joystick in one of four directions (forward, right, backward, or left) in order to trigger a short animation (8-9 seconds). One target appeared at a time at the top, right, bottom, or left sides of the screen, and only joystick inputs in the direction of the target produced the animation. We chose these four cardinal directions as an initial evaluation of a multi-dimensional cause-and-effect paradigm. A yellow star in the center of the screen served as the “cursor”, and moved toward the target when the animation was activated. We created the targets and corresponding animations using age-appropriate sprites (*i.e.,* monkeys, bubbles, musical instruments, and cars) that were recognizable by most children in our tested age range (Fig 2).

### Experimental setup

**Fig 3:**
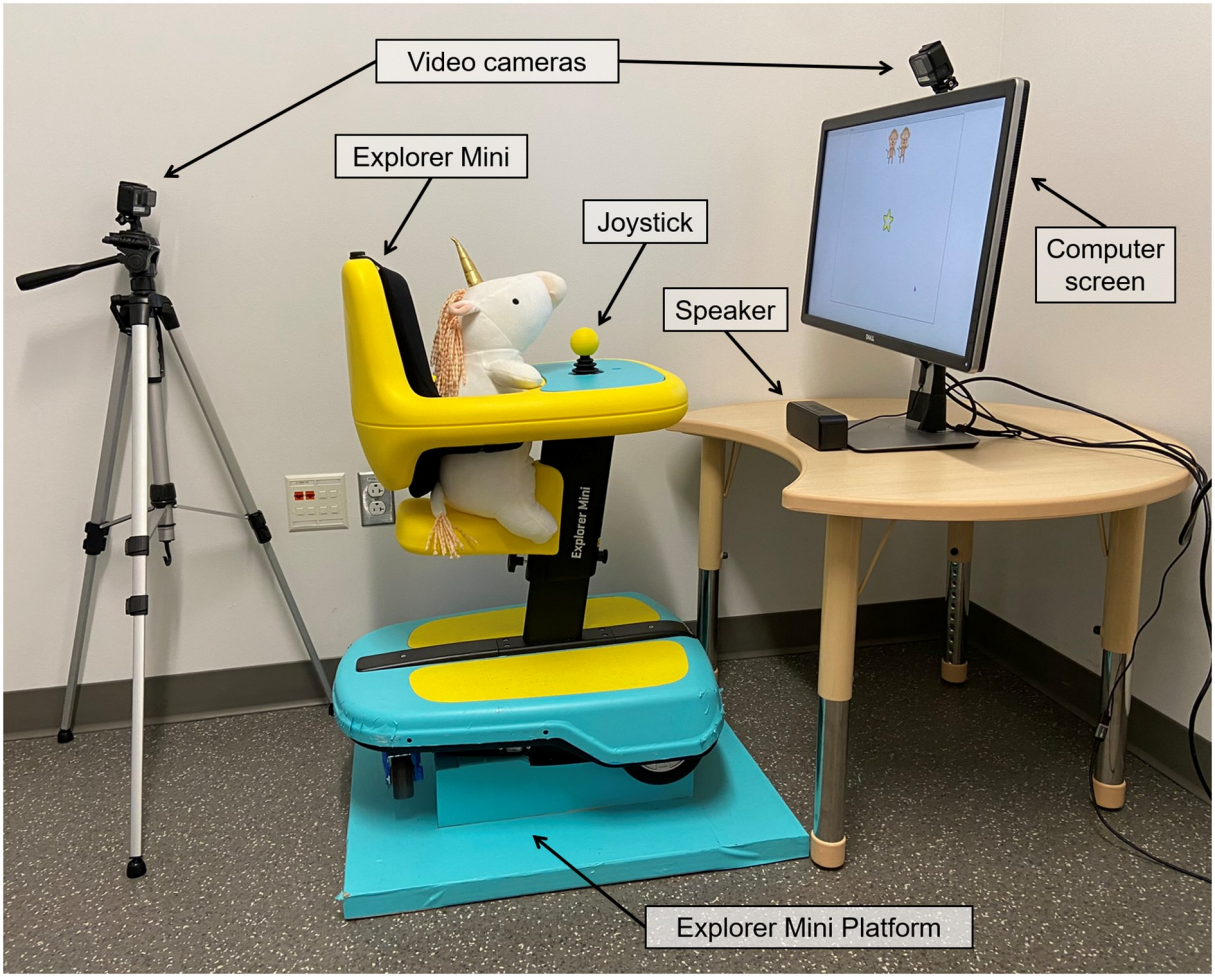
Experimental equipment and setup.

For our experimental platform, we used the Permobil Explorer Mini (Permobil AB, Timrå, Sweden), which is the only commercially available joystick-based mobility technology in the United States specifically designed for children under the age of three. We chose this platform because it features developmentally appropriate adjustable seating, and is bright, colorful, and looks fun and appealing to young children (Fig 3). The joystick itself is located at the midline with the bright yellow ball handle, and it was designed to provide appropriate sensory input experiences for very young children, as well as promote visual development, grasp and release, and bimanual midline manipulation. After the joystick has been moved, it will return to neutral when it is released. For this study, we elevated the Explorer Mini and secured it to a custom platform. Consequently, joystick inputs did not cause movement of the device, and instead were used to interact with the game on the screen.

We positioned the Explorer Mini on its platform in front of a small table (Fig 3). On the table, there was a computer monitor (screen dimensions: 60 cm wide × 34 cm tall) and a small external speaker. The screen was approximately 0.6 m in front of the child, positioned as close to eye-level as possible. Two video cameras (HERO5 Black, GoPro, San Mateo, CA) recorded the experiment from two different angles; one was mounted to the top of the computer monitor facing the child, and the second was mounted to a tripod behind the child, facing the screen. We performed the experiments in a small, quiet room with blank walls to minimize distraction.

### Joystick interface

**Fig 4:**
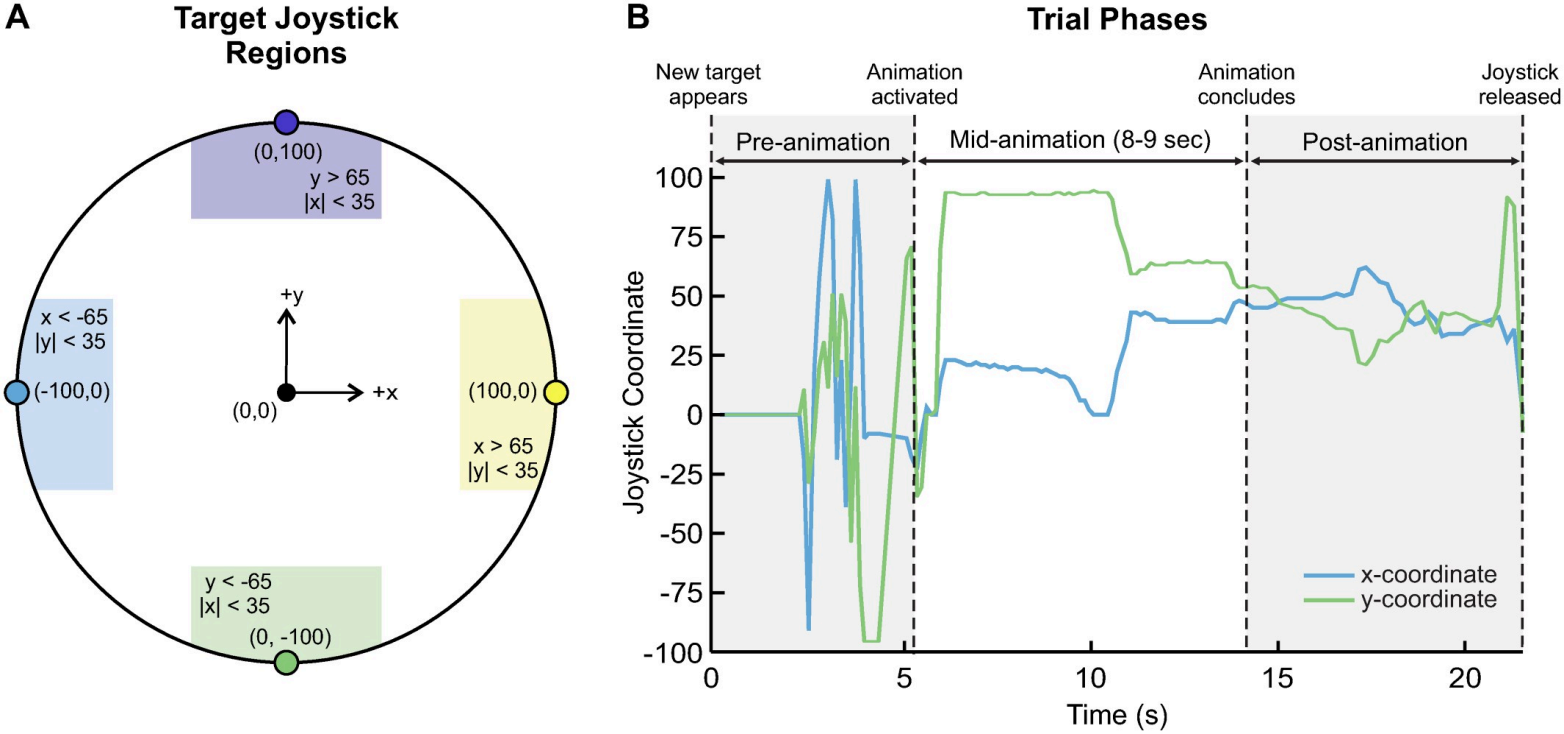
Description of the joystick interface. A: Joystick (*x*,*y*) coordinate system. Shaded target regions bound the area the joystick must enter to activate the game animation for each direction. Logical rules for (*x*,*y*) coordinates are written inside the shaded regions. B: Time-series graphical representation of the trial phases analyzed, using representative data from one trial.

We measured the child’s interaction with the joystick using custom software developed by collaborators at LUCI [43]. We used Python 3.10 to read joystick inputs in real time at approximately 100 Hz. We recorded real-time joystick inputs as (*x*, *y*) coordinates, where neutral was (0, 0) and *x* and *y* each ranged from -100 to 100. Through pilot testing, we determined an appropriate target region for the (*x*, *y*) joystick coordinates that should trigger the animations (Fig 4A). To trigger the animation, the x- or y-coordinate in the direction of the target had to exceed 65, and the other coordinate had to be less than 35. Custom Python code determined when the joystick was moved into these target regions and subsequently generated the appropriate series of key presses to trigger the Scratch animations.

### Experimental protocol

The game began with 8 “training” trials in which each target appeared twice in a row in the order top, right, bottom, left. During the training block, the experimenter explained how the child can use the joystick (typically referred to as “the yellow ball”) to make the animation play. The experimenter used a combination of verbal and physical prompts to teach the child how to play the game and reach the targets in different directions. One technique employed was to narrate the child’s current direction with the joystick, and suggest modifications to reach the target (*e.g.,*“You’re pushing forward! What happens if you pull towards you? Can you pull backward?”). The experimenter also offered physical prompts by pointing or demonstrating different directions, and provided hand-over-hand assistance when needed. The experimenter also taught the child to let go of the joystick after the animation finished in order to produce a new trial. The practice trials were not included in the analyzed data.

The game continued with 16 “test” trials. The first four test trials appeared sequentially in order (top, right, bottom, left) to reinforce the training the child just received. The next 12 test trials were presented in three blocks, each containing a random permutation of the four targets. This pseudo-randomization strategy was chosen over pure randomization of the remaining 12 trials to ensure that each target was evenly dispersed throughout the trials. During the test block, when a new target appeared, the experimenter announced the new target to bring the child’s attention to the screen (*e.g.,* “Do you see the green car?”) and gave the child approximately 10-15 seconds to explore and interact with the joystick. After 15 seconds, if the child hadn’t activated the animation, the experimenter gave a verbal prompt to remind the child they could control the game using the joystick (*e.g.,* “How can you use the yellow ball to make the cars drive?”). After 30 seconds, the experimenter gave directional prompts (*e.g.,* “You’re trying forward and backward, can you try side-to-side?”). After 45 seconds, the experimenter provided hand-over-hand assistance to activate the target while narrating their actions (*e.g.,* “We can use the yellow ball together to make the cars drive. We push the yellow ball to the side!”). After the child successfully activated the animation, the experimenter reinforced the child’s actions (*e.g.,* “You pushed to the side and made the cars drive!”). After the animation concluded, the experimenter gave the child approximately 5 seconds before reminding them they can reset the trial (*e.g.,* “How can we get a new one?”). After 10 seconds, the experimenter directly prompted the child (*e.g.,* “Can you let go to see a new one?”). We used these time increments to guide the protocol, but the experimenter used their discretion to give children more or less time to explore based on their demeanor, interaction with the joystick, and interest in playing the game.

Following the experiment, we used the video recording from the GoPro camera facing the child to identify which hand(s) the child used on the joystick to activate the animation each trial. We noted if the child used a different body part (*e.g.,* forearm, elbow, belly) to activate the animation with the joystick, and if this activation appeared accidental or intentional. We also identified when the experimenter provided hand-over-hand assistance to activate the animation, or if the experimenter activated the animation by themselves.

### Caregiver survey

After the child finished playing the game, the caregiver was asked to fill out a short survey. Caregivers were asked to report what they have observed about their child’s hand dominance/preference in daily life. The provided options were: 1) Strongly prefers left, 2) Shows some preference for left, 3) No clear preference/haven’t noticed, 4) Shows some preference for right, and 5) Strongly prefers right. Caregivers were also asked if their child regularly interacted with a touch-screen computer (*i.e.*, tablet or phone) and/or a non-touch-screen computer. If yes, they were asked to report how many minutes per day (on average) the child interacted with the devices, and asked to describe the activities the child did with the device (Playing games, Watching something, Video chatting, or Other).

### Data analysis

In this study, we quantified how children played the game with the joystick using performance, accuracy, time, path length, and hand use metrics. Only trials in which the child used one or two hands to activate the animation by themselves (*i.e.,* successful trials) were included in the data analysis. All analyses were performed using MATLAB R2021b (MathWorks, Natick, MA).

Informed by prior literature in this space, we investigated how age and gender affected children’s performance in the game, as well influenced the way they interacted with the joystick. Further, we were interested in how these metrics varied between different sub-phases of the task in order to inform future assistive algorithms. To this end, we divided each trial in three phases (Fig 4B). The pre-animation phase was defined from the moment a new target appeared to when the child successfully activated the animation. The mid-animation phase was defined as the duration that the animation was playing. The post-animation phase was defined from the moment the animation concluded until the child released the joystick back to neutral.

To analyze the number of successful trials, we utilized a one-way ANOVA, with age group as a fixed factor. We subsequently used a Tukey-Kramer *post-hoc* test, with significance level of 0.05. To analyze the time and path length metrics, we used linear mixed-effects models (LMEMs) with age as a continuous fixed factor, gender as a categorical fixed factor, and an interaction term. We used all successful trials completed by all participants to build these models, and a random intercept for each participant was included in each model. For a particular metric, if the interaction term between age and gender was not significant (at a significance level of 0.05), we re-ran the model using only age and gender as fixed factors.

## Results

### Number of successful trials

**Fig 5:**
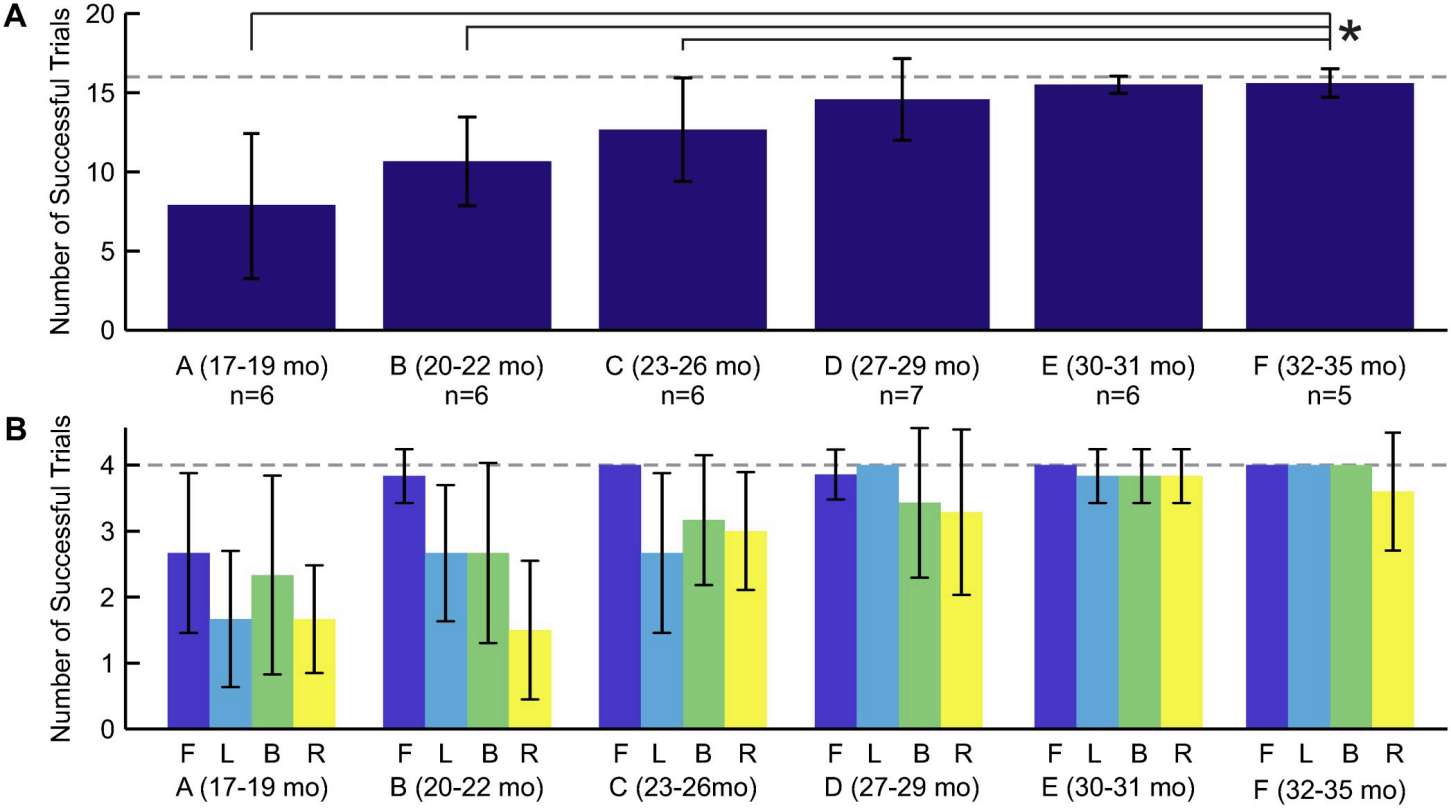
Analysis of successful trials. A: Dark blue bars depict the inter-participant mean number of successful trials for each age group (A-F). The age range (in months) and number of participants (n) are shown for each group, and can also be found in Table 1. Black error bars represent 1 SD. The gray dashed line indicates the maximum number of trials each child performed (16 trials). Black brackets with an asterisk denote significantly different means between groups (*α* <.05). B: The mean number of successful trials per age group, categorized by target direction (F = forward, L = left, B = backward, R = right). Black error bars show 1 SD. The gray dashed line indicates the maximum number of trials children performed in each direction (4 trials).

Successful trials were defined as the number of test trials (out of 16) that a child activated the animation using one or both hands on the joystick. There was a significant effect of age on the number of successful trials (ANOVA: F(5,30)=6.95, *p* < 0.001). The oldest age group (F) completed significantly more trials than each of the three youngest age groups (Tukey-Kramer: A, *p*=0.001; B, *p* < 0.001; C, *p*=0.002) (Fig 5A). Fig 5B depicts the number of successful trials classified by target direction. For targets in the forward direction, the youngest age group averaged 2.8 successful trials, while all other age groups averaged >3.8 successful trials (out of 4). For all other target directions, older children trended toward more successful trials than younger children.

### First movement direction

**Fig 6:**
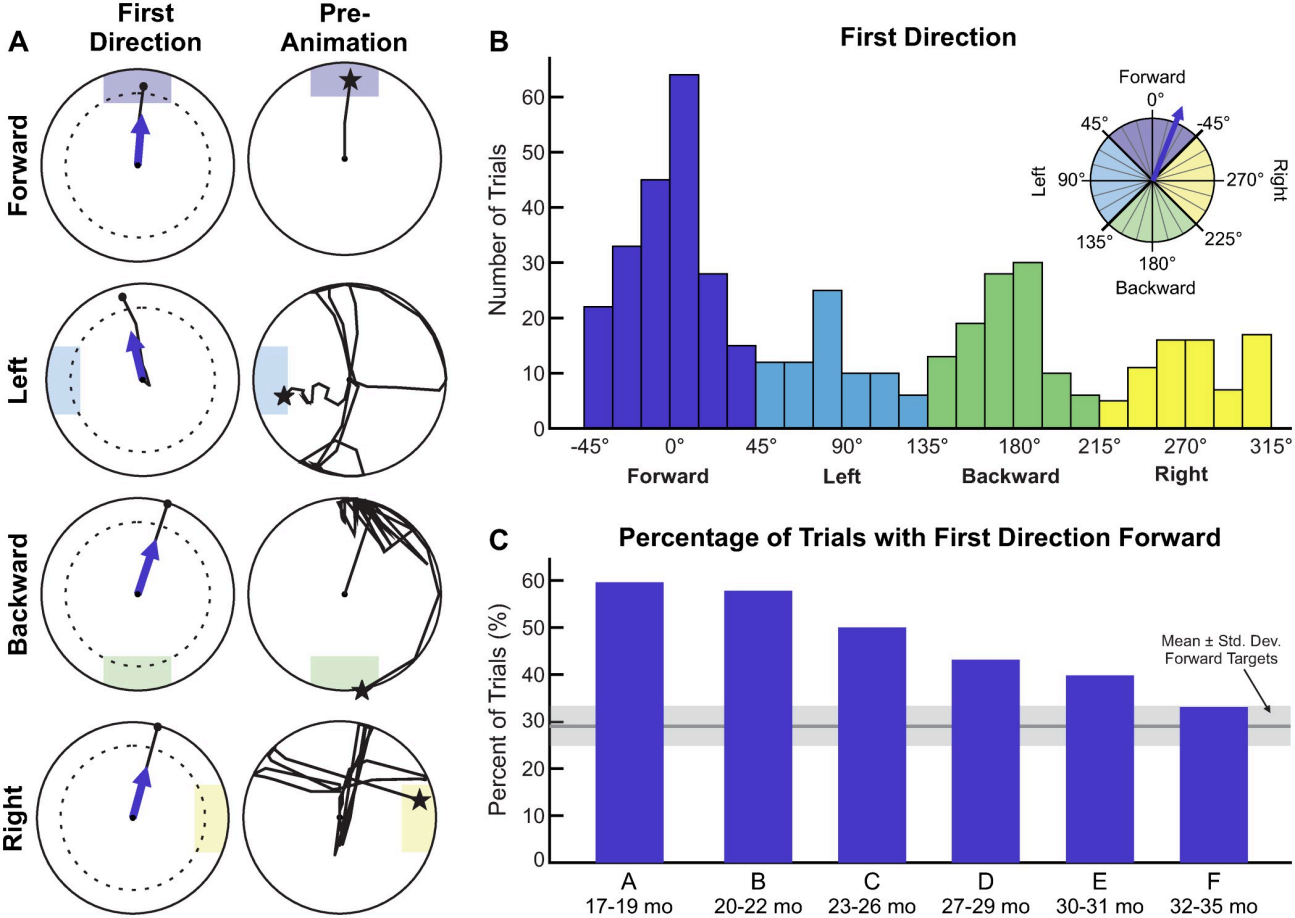
Analysis of first direction. A: Representative trials with a forward first direction. Rows show trials with a forward, left, backward, or right target. Both the first direction (left column) and pre-animation phase (right column) are shown. The shaded areas indicate the target region that triggers the animation. In the first column, the dotted line represents the radial threshold (75) that is considered a first movement, and the purple arrow indicates the direction of the first movement vector. In the second column, the star shows the joystick position when the animation is activated. B: Histogram of first direction angle (*θ*_*f*_), pooled across all participants. The first direction was classified into one of four quadrants based on its angle. C: The solid purple bars show the percentage of all pooled trials per age group (A-F) that were classified as forward. Group definitions can be found in Table 1. The gray line and shaded region in the background shows the mean ± 1 SD of the percent of trials that had forward targets, calculated across age groups.

We identified the first direction that children used when initiating movement with the joystick. The first direction vector was defined as the line connecting two points: joystick neutral at (0, 0), and the first data point (*x*_*f*_, *y*_*f*_) that the joystick crossed a radial threshold of 75. We calculated the angle of this vector (*θ*_*f*_) with respect to vertical, with + *θ* defined counterclockwise from the positive y-axis. We segmented the joystick’s circle into quadrants to classify the first direction as forward, left, backward, or right, based on the magnitude of the first direction vector angle (Fig 6B).

Fig 6A shows four representative trials in which the first direction was classified as forward, one for each target direction. In these trials, children eventually reached the target through exploration in the pre-animation phase, but their first joystick movement was in the forward direction.

We found that children were more likely to move the joystick forward first than any other direction (Fig 6B). Pooling successful trials from all participants (460 trials), we generated a distribution of first direction vector angles (*θ*_*f*_), segmented by angular increments of 15 degrees. Forward comprised 45.6% of all first joystick directions, compared to 16.3% left, 23.2% backward, and 14.3% right.

Pooling successful trials for each age group (A: 47 trials, B: 64 trials, C: 76 trials, D: 102 trials, E: 93 trials, F: 78 trials), we calculated the percentage of trials with forward first directions(Fig 6C). This proportion decreased with age, with the youngest children using a forward first direction 59.6% of the time, and the oldest children using a forward first direction 33.3% of the time. Across age groups, the mean (SD) percentage of all targets that were in the forward direction was 29.6% (4.2%).

### Accuracy of first direction

**Fig 7:**
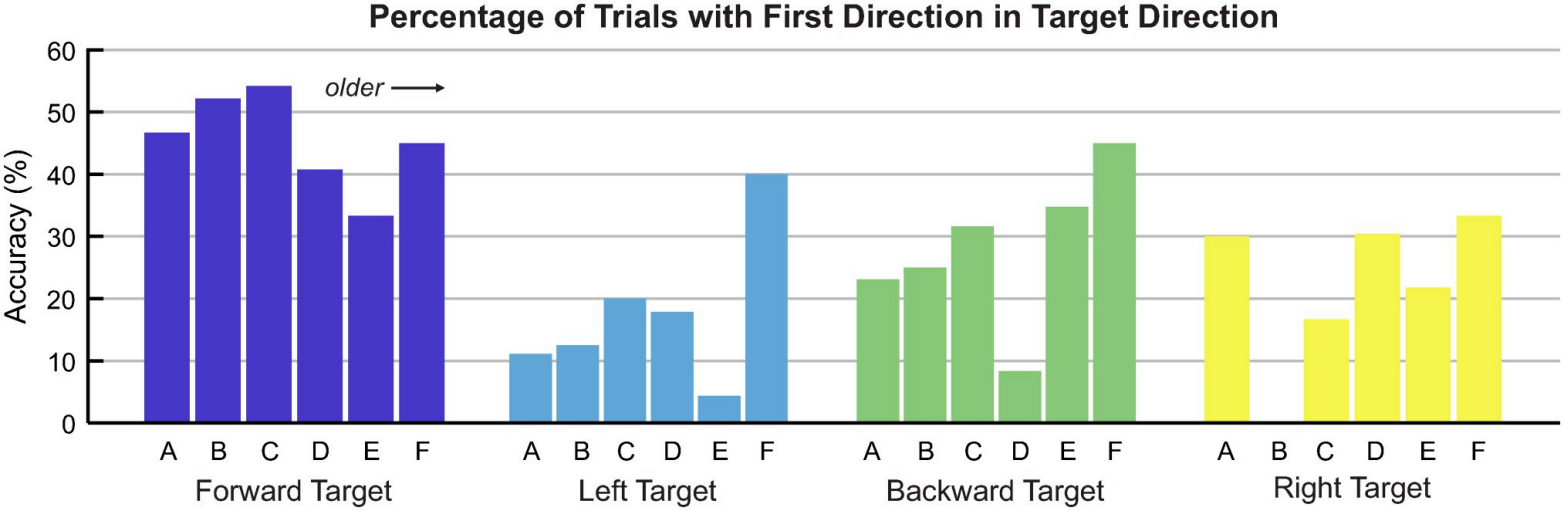
Analysis of accuracy. Accuracy was defined as the percentage of trials where first direction and target direction were aligned. Solid bars depict the accuracy for each target direction and age group, and data were pooled across all participants within an age group. Age groups (A-F) are defined in Table 1, where A is the youngest and F is the oldest.

We calculated accuracy as the percentage of trials in which the first direction matched the target direction. For this analysis, we pooled data from all participants per age group. Children were most accurate in the forward direction, with all but group E surpassing 40% accuracy (Fig 7). In the forward direction, younger age groups (A-C) were more accurate than older age groups (D-F). Across all directions, the oldest age group (F) demonstrated relatively consistent accuracy, between 33% and 45%, and there was a trend toward increased accuracy with increased age.

### Time metrics

**Fig 8:**
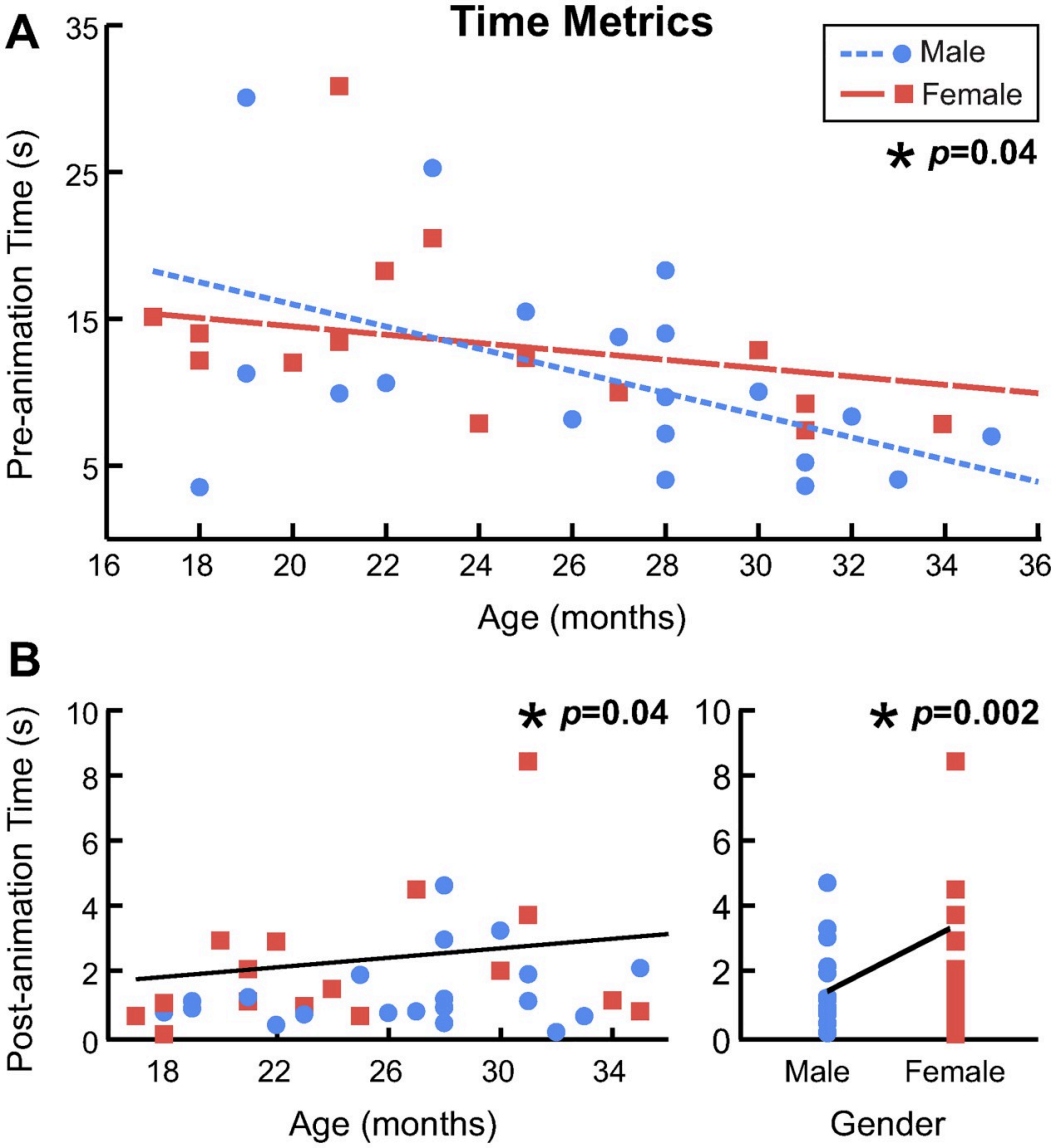
Analysis of time metrics. A: Mean pre-animation time for each participant. The blue circles are boys and the red squares are girls. The dotted blue line and dashed red line show the expected relationship between pre-animation time and age for each gender. The asterisk and *p*-value indicate a significant interaction effect. B: Mean post-animation time for each participant. The solid black lines show the expected relationship between pre-animation time and age (left), and gender (right). The asterisks and *p*-values indicate a significant fixed effect.

The time metrics captured how long it took for children to perform certain joystick actions. Participants’ pre-animation time (*i.e.,* how long it took the child to move the joystick into the target region) decreased with age, and there was a significant interaction between age and gender (Fig 8A). For girls, the linear mixed effects model (LMEM) predicted that pre-animation time decreases by 0.29 seconds per month of age; for boys, the model predicted a decrease of 0.76 seconds per month (Table 2). According to the slopes of the model, we would expect 35-month-olds to activate the animation 5.1 and 13.6 seconds faster than 17-month-olds, in girls and boys, respectively.

**Table 2:** Parameter estimates from linear mixed effects models with age, gender, and interaction fixed effects.

	*p*-value	Slope		Intercept	
		Estimate [95% CI]		Estimate [95% CI]	
	Interaction	Boys	Girls	Boys	Girls
Pre-Animation Time (s)	0.04	-0.76	-0.28	31.09	20.18
		[-1.5, -0.01]	[-0.58, 0.01]	[10.84, 51.35]	[12.27, 28.09]

Participants’ post-animation time (*i.e.,* how long it took the child to release the joystick after the animation concluded) significantly increased with age (Fig 8B). The LMEM predicted that post-animation time increases by 0.07 seconds per month of age (Table 3). Thus, we would expect 17-month-olds to release the joystick 1.37 faster than 35-month-olds. The LMEM also revealed a significant effect of gender on post-animation time, with boys expected to release the joystick 1.1 seconds faster than girls (Fig 8C) (Table 3).

**Table 3:** Parameter estimates from linear mixed effects models with age and gender fixed effects.

	*p*-value	Age	*p*-value	Gender
	Age	Estimate [95% CI]	Gender	Estimate [95% CI]
Post-Animation Time (s)	**0.04**	**0.07 [0, 0.14]**	**0.002**	**-1.1 [-1.8, -0.4]**
Pre-Animation Path Length	**0.001**	**-58.9 [-94.12, -23.64]**	0.73	61 [-292.97, 415.03]
Mid-Animation Path Length	0.82	4.3 [-34.62, 43.19]	0.38	180.3 [-226.4, 587.1]
Post-Animation Path Length	0.34	25.2 [-26.5, 76.9]	0.34	-263 [-801.4, 275.5]

**Bold** text indicates a significant effect

### Cumulative path length

**Fig 9:**
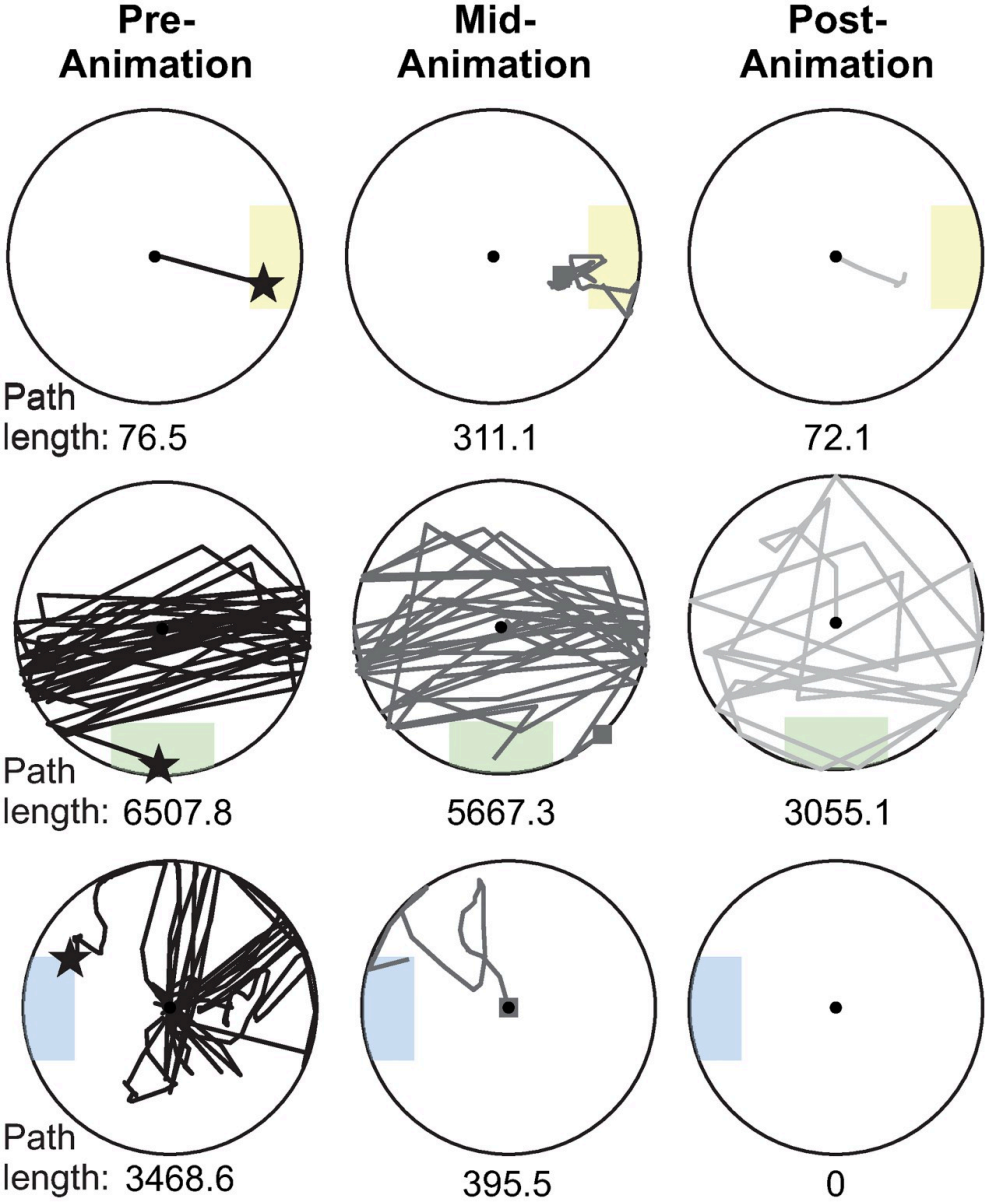
Representative trials for three participants. One participant is shown per row, with cumulative path length calculated for the pre-animation (left column), mid-animation (middle column), and post-animation (right column) phases. The cumulative path length for each trajectory is shown at the bottom of each circle. Shaded regions indicate the target area. For the pre-animation phase, the star indicates the joystick position when the animation was activated. For the mid-animation phase, the square shows the joystick position when the animation concluded. For the post-animation phase, the participant returned the joystick to the center of the circle.

Cumulative path length quantified how much the child moved the joystick in Cartesian (*x*, *y*) space, and can be used as a metric of efficiency. Path length was defined as the distance between two consecutive data points recorded from the joystick, calculated by: $\sqrt{({\left(x_{i}-x_{i-1}\right)}^{2}+{\left(y_{i}-y_{i-1}\right)}^{2}}$, where (*x*_*i*_, *y*_*i*_) is the joystick position at the current time *t*_*i*_ and (*x*_*i*−1_, *y*_*i*−1_) is the joystick position at the previous time step *t*_*i*−1_. We calculated the cumulative path length (the sum of all path lengths within the trial phase) for the pre-, mid-, and post-animation phases. Depending on the speed of movement and the sampling rate of the joystick signal, the most efficient path to the target is approximately 65-110. Fig 9 illustrates representative trials for three participants during each phase of the trial, with cumulative path length calculated for each joystick trajectory.

**Fig 10:**
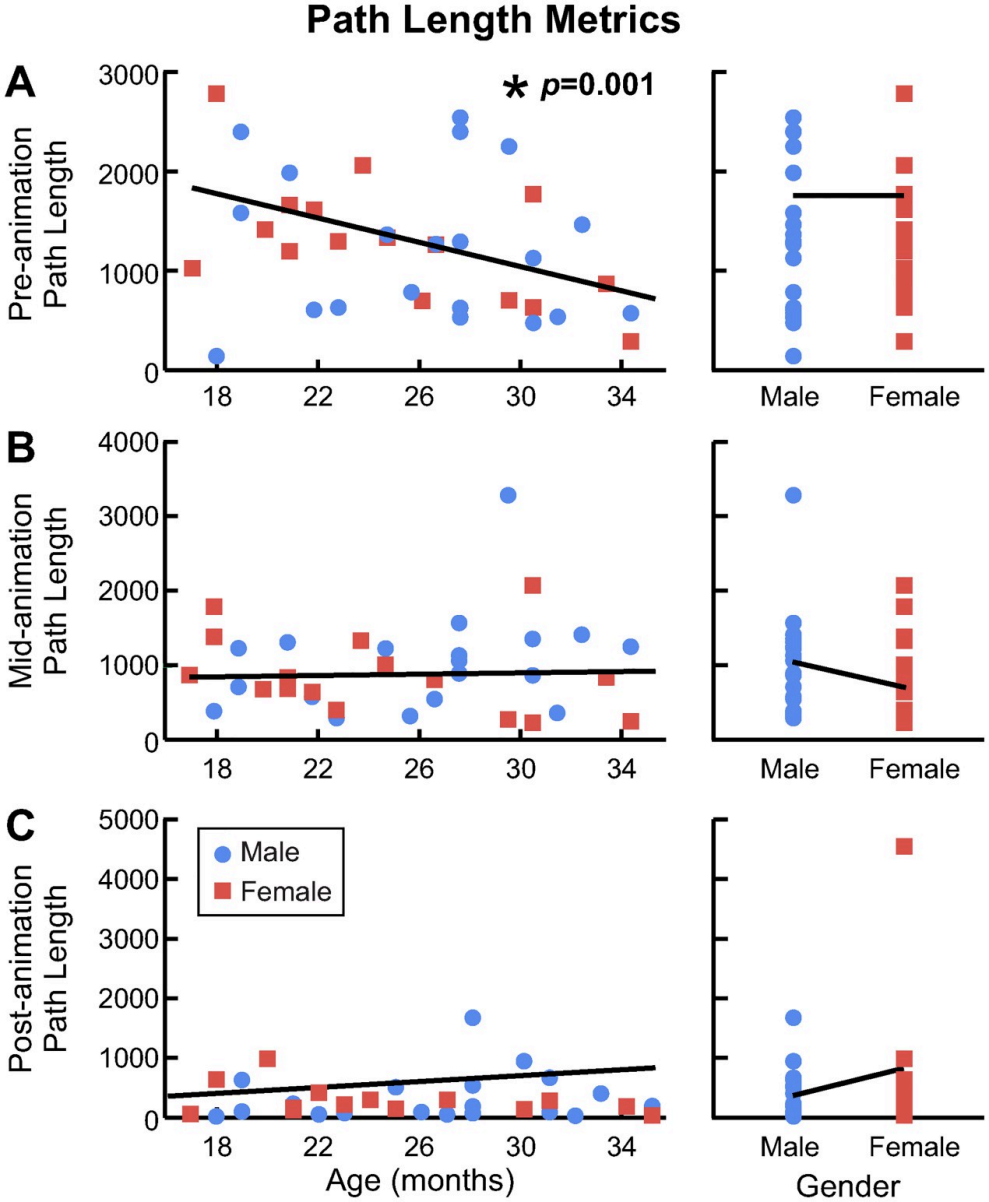
Analysis of path length metrics. A: Mean pre-animation path length, B: Mean mid-animation path length, and C: Mean post-animation path length for for each participant. The blue circles are boys and the red squares are girls. The solid black lines show the expected relationship between path length and age (left), and gender (right). The asterisk and p-value indicate a significant fixed effect.

Pre-animation cumulative path length significantly decreased with age, which indicates that older children completed the task more efficiently (Fig 10A). The LMEM predicted that participants’ path length decreases by 71.5 units per month of age (Table 3). Therefore, we would expect that the path to reach the target was 1287.7 units shorter for 17-month-olds than 35-month-olds. There were no significant effects of age, gender, or their interaction on mid-animation or post-animation cumulative path length (Table 3) (Fig 10B and 10C).

### Hand use

**Fig 11:**
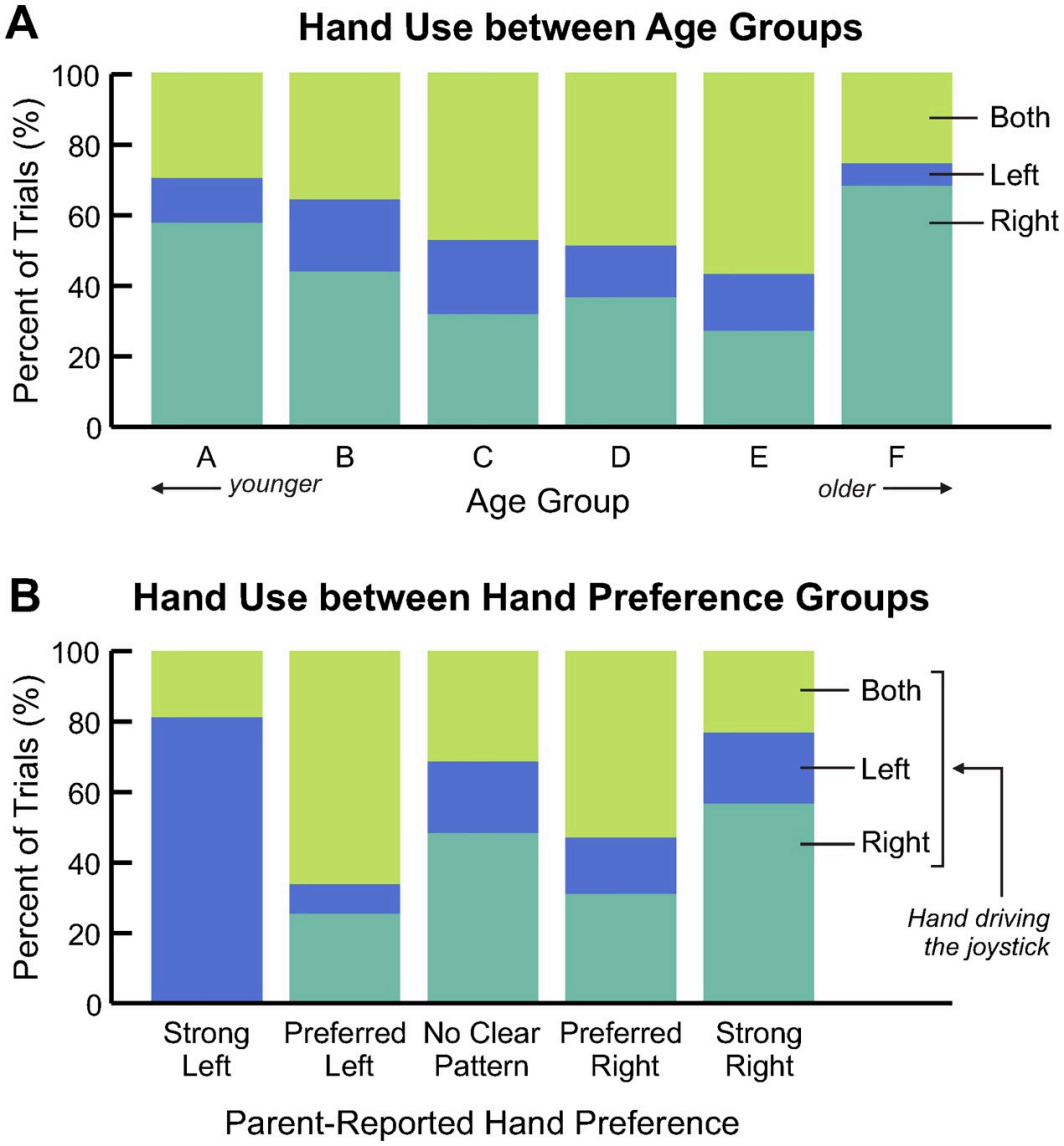
Analysis of hand use. Percent of trials in which children used both hands (top stacked bar), left hand (middle stacked bar), or right hand (bottom stacked bar) on the joystick to activate the animation. A: Mean data are shown for each age group (A-F), where A is the youngest and F is the oldest; group definitions can be found in Table 1. B: Mean data are shown for each group of parent-reported hand preference on the post-experiment survey.

To understand how toddlers use their upper limbs to interact with the joystick, we used the recorded video to determine which hand (left, right, or both) children used on the joystick. Over the four youngest age groups (A-E), there was a trend toward higher bimanual joystick use in older children (Fig 11A). Similarly, we saw a trend towards lower right hand use in older children, and left hand use remained relatively constant. In the oldest age group, participants used their right hand on 70% of trials.

Hand dominance or preference in toddlers begins to emerge within the child’s first two years [44], but it can not be reliably assessed until children are 4-6 years old [45]. For this reason, we relied on feedback from the parents about their observations of their child’s early hand preference. On the post-experiment survey, 1 parent reported their child strongly prefers left, 5 reported the child has a preference for left, 8 reported no clear pattern, 9 reported the child has a preference for right, and 10 reported the child strongly prefers right. Three parents declined to respond. The one child whose parent reported they demonstrated a strong preference for left used their left hand on the joystick during 80% of successful trials (Fig 11B). The eight children whose parents reported they demonstrated a strong preference for right used their right hand an average of 57% of successful trials.

### Reported technology use

When asked if their child regularly interacts with a touch-screen computer, 25 of the 35 caregivers who responded reported ‘yes’ and 10 responded ‘no’. For the 25 who reported ‘yes’, they reported the child uses the touch screen computer for an mean (SD) of 22.1 (23.6) minutes per day. The most common ways caregivers reported their children were using a touch-screen computer was for ‘video chatting’, ‘watching something’, and ‘playing games’. Only one caregiver reported that their child regularly uses a non-touch-screen computer, stating that their child used a LeapFrog computer with a keyboard interface for playing games.

## Discussion

Understanding joystick use in children under the age of three has important implications for the design and deployment of technologies that can support toddlers in exploration and play. To this end, our study quantitatively characterized toddlers’ initial interactions with a joystick interface as they played a simple cause-and-effect game on a computer screen. While performance, interest, and attention varied between individual children, we identified several overall trends in time, path length, and preferred movement direction metrics with respect to age and gender. Synthesizing the results from this study, we discuss our findings as they relate to three key themes: 1) forward first, 2) the curse of directionality, and 3) toddlers as technology users. Each of these themes describe features of children’s joystick interactions that have important implications for the design of developmentally appropriate technologies.

### Forward first

The most surprising finding was the frequency in which children went ‘forward first’, or initiated joystick movement in the forward direction. It is especially interesting to see that although children (especially the youngest) moved forward first substantially more than any other direction, they only achieved 30-55% accuracy on forward targets. This indicates that moving forward first was not entirely a conscious decision that children made based on the target they saw on the screen (more discussion on this point the subsequent section).

Developmental age and emerging fine motor skills may have influenced participants’ forward first tendencies. For example, most nondisabled children ages 18-23 months can push and pull objects easily, but more complex hand/wrist coordination (*e.g.,* unscrewing a lid on a jar) is not usually achieved until closer to 29 months [46]. Therefore, we may expect for a similar neutral hand position (hand on top or slightly behind the ball), a forward push is an easier and more intuitive motion for younger children than backwards or side-to-side. While we did not observe a clear relationship between the hand used on the joystick and age or reported hand preferences (Fig 11), it is possible that a combination of which hand is driving the joystick, the hand position, and the child’s developing hand preference may influence first movement directions. Additional research on the precise limb coordination patterns and biomechanics of joystick use is required to further understand this phenomenon. Given the participants’ young age, it is also likely that the cognitive developmental processes that underlie goal-directed behavior are still emerging [47]. Future research could more deeply investigate these outcomes from a developmental psychology perspective.

In the context of toddlers using joystick-controlled powered mobility devices, similar (although less pronounced) trends in preferred joystick direction were observed in 33 toddlers with disabilities driving the Explorer Mini for the first time [48]. Within two 15-minute play sessions, 82% of children used the joystick to move the device in the forward direction, compared with 70% backwards, 61% in circles, 61% right and 48% left. For children driving the Explorer Mini, moving the joystick primarily in the forward direction has advantages for goal-directed mobility and participation in play with peers. However, our findings suggest that children this age may default to using the forward direction. Thus, future studies should explore assistive control algorithms that gently assist with navigation, obstacle avoidance, or steering.

### The curse of directionality

The majority of participants in the study demonstrated that they understood the basic cause-and-effect premise of the game: moving the joystick makes the animation play. However, children’s directional accuracy was quite low for all age groups and target directions (Fig 7). This indicates that most participants did not understand the relationship between the *direction* of their joystick movement and the location of the target on the screen. As a result, children explored broadly with the joystick in order to successfully activate the animation.

Evidence of this strategy can be seen by inspecting participants’ joystick trajectories. With the short moment arm of the Explorer Mini joystick, most participants were able to quickly and smoothly move the joystick to the perimeter in many directions (examples are shown in Figs 6 and 9). Furthermore, the task did not require precise positioning of the joystick (the target regions were relatively large) and it was not possible to overshoot the target. Yet, even the oldest children had difficulty completing the task efficiently, moving the joystick 7-10 times farther than the most efficient path to the target (Fig 10). Examining the joystick trajectories, it becomes clear that these inefficiencies are more likely a result of children not knowing the direction to move the joystick to activate the animation than from difficulty positioning the input device itself.

One of the reasons that children in the study may have had difficulty understanding the directionality of the game was the mismatch between the joystick’s action plane (horizontal) and the plane of the screen (vertical). Successfully performing the task required that children not only understand that different joystick actions are required for different targets on the screen, but also associate “forward” with “top” and “backward” with “bottom”. Preliminary visual-spatial skills, or the ability to understand relations between objects and spaces, have been measured in children as young as four months old, but the refinement and development of these skills continue well through adolescence and even into adulthood [49]. Therefore, it is likely that this transformation was too challenging for children this young to intuitively understand or figure out on their own through exploration.

This action plane mismatch exists for a large majority of joystick- and mouse-based tasks (*e.g.,* using a computer, playing video games, operating a powered wheelchair). Therefore, teaching children to understand this relationship from a young age may set them up to successfully use a variety of input interfaces in the future. There are several strategies that could be employed to teach young children how to use the joystick in front of them to interact with the world around them. In our study, we did not use a cursor that responded proportionally to real-time joystick inputs in an effort to keep the task as simple as possible for young children, but future studies should explore if visual proportional feedback helps toddlers learn the task. To support early learning, targets could also appear simultaneously on the computer screen and on the tray through the use of LEDs or high-tech visual tray displays to reinforce associations between joystick and screen directions. As toddlers learn through a combination of sensory inputs, haptic and audio feedback modalities should also be explored.

Lastly, children may be able to learn directional cause-and-effect by using a joystick to physically move their body through space. A recent study reported that 6-month old pre-locomotor nondisabled infants demonstrated significant improvements in the visual-spatial skill of mental rotation after locomotion training using a baby walker [50]. This indicates that self-initiated mobility may influence young children’s use of computers and technology, which has major implications for research in pediatric assistive devices and toddler-computer interaction.

### Toddlers as technology users

Our results demonstrate that children’s performance using a joystick for computer interaction improves with age, as has been shown in numerous studies evaluating children using a variety of input modalities [21, 22, 24, 25, 27]. Yet, it is notable that linear trends in the number of successful trials (Fig 5), the time to activate the animation (Fig 8), and the path length to the target (Fig 10) emerged over an age range of only 18 months, and in children so young.

Considering the time-based metrics, we observed that older participants activated the animation significantly faster than younger participants, and that the effect of age varied between genders (Fig 8A). Our model predicts that, at 17 months old, boys will perform the task slightly slower (by 3 seconds) than girls. But as age increases, girls’ pre-animation time decreases only slightly, while boys’ pre-animation time decreases more substantially. By the time children are 35 months old, the model predicts that boys will outperform girls by nearly 6 seconds. Research investigating gender differences in computer task performance has been mixed, with some studies reporting that 3-year-old boys were more proficient users [30] and others finding no difference between genders [24]. However, it is very difficult to draw comparisons between studies that test children of different ages performing different tasks with different input devices. Research has consistently found gender differences in children’s visual-spatial skills (e.g., [51]), which may explain why boys with stronger visual-spatial skills have been shown to be more proficient computer users. By contrast, a recent study documented no difference in visual-spatial skills between genders in two-year-olds [49], so it is not clear exactly when or whether these differences emerge. We also observed a significant increase in participants’ post-animation time as children aged. Although the effect of age on post-animation time is small (expected increase of 1.3 seconds from 17- to 35-months), it is possible that older participants continued moving the joystick after the animation concluded out of boredom or lack of attention. This highlights the importance of designing age-appropriate, engaging computer tasks for toddlers.

It is intriguing to consider that the way toddlers in this study initially interacted with the joystick (*i.e.,* forward first) could have emerged based on their prior experiences with technology. Another way to interpret the forward first behavior is that pushing the joystick forward is moving it *toward* the screen. It is possible that this pattern could be attributed to our participants’ mental model of how to interact with technology, based on their previous experiences. As reported by caregivers, the majority of children in our study (71.4%) regularly interacted with a touch screen phone or tablet, while only one child regularly interacted with a non-touch screen computer (a LeapFrog computer with a keyboard interface). Researchers posit that direct interfaces, such as touch, can be learned naturally and through observation, while indirect interfaces, such as joysticks, need to be learned through observation, experimentation, and processing of equivalence [34, 52]. Therefore, it is possible that the participants’ intuition was to move their body (and the joystick) toward the screen to generate a computer action, because they had not yet been exposed to indirect interfaces. This may be especially true for the youngest children, who would have also had fewer opportunities to observe people around them using using non-touch computers. During the experiment, we observed that several children tried to reach forward to touch the computer screen while playing the game, which provides anecdotal support for this idea.

### Limitations

This research represents a first-step toward quantifying joystick-based interactions and enabling additional options for toddlers to interact with their environment. In this work, we designed a cause-and-effect game that had targets in four directions: forward, backward, left, and right. We chose these directions because they are commonly experienced during everyday computer and game interaction, but future work should seek to understand the impact that the number and complexity of targets has on performance. There still remain many domains to be examined in future research including task space, control scheme, and other device-specific factors. While this study is an important first step toward understanding toddler-joystick interaction as a function of age and developmental stage, all the participants in our study were typically-developing children with no known mobility challenges.

We designed this study to intersect with the prior literature on switch and joystick use among toddlers. Thus, we prioritized child comfort, engagement, and fun through the research process. However, this did reduce the total number of trials, repeatability, and sensitivity analyses that could be integrated into our protocol. Further, while we tried to standardize our verbal interactions with the child, we recognize there are always variations in verbal coaching that are highly dependent on the child-experimenter and caregiver interactions. We also analyzed gender in this work to compare and align with prior research, but recognize that categorization and conceptualization of gender at this age can be unclear. As such, the observed gender effects could be due to other, unmeasured factors relating to the task or environment. Our caregiver survey only asked about the participants’ history of interaction with touch-screen and non-touch-screen computers. However, there are many other factors that could influence the way children perform the task, such as the types of toys they typically play with, how much they are exposed to a parent, caregiver, or older sibling using technology, or their cognitive developmental timeline [47, 53]. These factors should be explored in future research.

### Future directions

The novel experimental paradigm we described in this work—using the Explorer Mini joystick for real-time computer use—opens up a number of exciting avenues for future research and technology development. One such area of interest is investigating the intersections between joystick use for computer interaction and mobility using a common, integrated platform for both tasks. To understand and design effective assistive technologies for children with disabilities, we are motivated to determine how exploration, play, and enriching computer use experiences can be simultaneously introduced to young children to foster positive growth and development. Furthermore, we seek to quantify how toddlers incorporate feedback from different sensory modalities (*e.g.,* visual, audio, or haptic) and modify their physical interaction with an input device. Crucially, the next phase of this work will expand the participant pool to include toddlers with disabilities, who may utilize different interaction techniques and have different motivations for using technology.

## Conclusion

For toddlers, interacting with technology can provide access to learning, exploration, and play. Given the ubiquity of modern touch screen interfaces and the relative ease with which toddlers can use these devices, a large volume of research effort has been dedicated to studying these interfaces with young children. Despite their popularity, it is important to remember that touch screen interfaces may not be appropriate for all children or all tasks. Therefore, joystick-based interfaces should also be considered for play technologies like video games or motorized toys. In this paper, we characterized how nondisabled toddlers used a joystick to interact with a cause-and-effect computer program. We found that during toddlers’ initial experiences with a joystick interface, they demonstrated a strong preference for moving the joystick forward first, especially amongst the youngest children. Older children performed the task better than younger children, and linear trends in the number of successful trials, time to activate the program, and the path length to the target emerged with respect to age. Importantly, we found that toddlers’ challenges and/or inefficiencies related to performing the task were more likely attributable to their lack of understanding of directional cause-and-effect, rather than to their difficulty using the joystick interface. The findings from this study can be used as a starting point to inform the design of assistive algorithms and inclusive joystick interfaces that are intuitive and easy to learn for children this age. In the future, expanding this work to understand how toddlers with disabilities use a joystick for both mobility and computer use will generate necessary insights on how to provide equitable access to technology for children during critical developmental periods.

## References

[1] McCarrick K, Li X, Fish A, Holtrop T, Bhavnagri NP, Stanton B, et al. Parental involvement in young children’s computer use and cognitive development. NHSA DIALOG. 2007;10(2):67–82. doi: 10.1080/15240750701436410

[2] Li X, Atkins MS, Stanton B. Effects of home and school computer use on school readiness and cognitive development among Head Start children: A randomized controlled pilot trial. Merrill-Palmer Quarterly (1982-). 2006; p. 239–263. doi: 10.1353/mpq.2006.0010

[3] Li X, Atkins MS. Early childhood computer experience and cognitive and motor development. Pediatrics. 2004;113(6):1715–1722. doi: 10.1542/peds.113.6.1715 15173496

[4] Plowman L, Stephen C. Children, play, and computers in pre-school education. British Journal of Educational Technology. 2005;36(2):145–157. doi: 10.1111/j.1467-8535.2005.00449.x

[5] Papalia DE, Olds SW, Feldman RD. A child’s world: Infancy through adolescence. McGraw-Hill New York; 1990.

[6] Byrnes JP. Piaget’s cognitive-developmental theory. Encyclopedia of Infant and Early Childhood Development. 2008;87:543–552. doi: 10.1016/B978-012370877-9.00122-5

[7] Piaget J. Science of education and the psychology of the child. Orion; 1970.

[8] Flavell JH. Cognitive development: Past, present, and future. Developmental Psychology. 1992;28(6):998–1005. doi: 10.1037/0012-1649.28.6.998

[9] Judge SL. Computer applications in programs for young children with disabilities: Current status and future directions. Journal of Special Education Technology. 2000;16(1):29–40. doi: 10.1177/016264340101600103

[10] Chantry J, Dunford C. How do computer assistive technologies enhance participation in childhood occupations for children with multiple and complex disabilities? A review of the current literature. British Journal of Occupational Therapy. 2010;73(8):351–365. doi: 10.4276/030802210X12813483277107

[11] Judge S, Floyd K, Wood-Fields C. Creating a technology-rich learning environment for infants and toddlers with disabilities. Infants & Young Children. 2010;23(2):84–92. doi: 10.1097/IYC.0b013e3181d29b14

[12] Tam C, Schwellnus H, Eaton C, Hamdani Y, Lamont A, Chau T. Movement-to-music computer technology: a developmental play experience for children with severe physical disabilities. Occupational Therapy International. 2007;14(2):99–112. doi: 10.1002/oti.227 17623382

[13] Østensjø S, Carlberg EB, Vøllestad NK. The use and impact of assistive devices and other environmental modifications on everyday activities and care in young children with cerebral palsy. Disability and Rehabilitation. 2005;27(14):849–861. doi: 10.1080/09638280400018619 16096237

[14] Dicarlo CF, Banajee M. Using voice output devices to increase initiations of young children with disabilities. Journal of Early Intervention. 2000;23(3):191–199. doi: 10.1177/10538151000230030801

[15] Romski M, Sevcik RA. Augmentative communication and early intervention: Myths and realities. Infants & Young Children. 2005;18(3):174–185. doi: 10.1097/00001163-200507000-00002

[16] Light J, Drager K. AAC technologies for young children with complex communication needs: State of the science and future research directions. Augmentative and Alternative Communication. 2007;23(3):204–216. doi: 10.1080/07434610701553635 17701740

[17] Lynch A, Ryu JC, Agrawal S, Galloway JC. Power mobility training for a 7-month-old infant with spina bifida. Pediatric Physical Therapy. 2009;21(4):362–368. doi: 10.1097/PEP.0b013e3181bfae4c 19923977

[18] Agrawal SK, Chen X, Ragonesi C, Galloway JC. Training toddlers seated on mobile robots to steer using force-feedback joystick. IEEE Transactions on Haptics. 2012;5(4):376–383. doi: 10.1109/TOH.2011.67 26964134

[19] Galloway JC, Ryu JC, Agrawal SK. Babies driving robots: Self-generated mobility in very young infants. Intelligent Service Robotics. 2008;1(2):123–134. doi: 10.1007/s11370-007-0011-2

[20] Hourcade JP, Bederson BB, Druin A, Guimbretière F. Differences in pointing task performance between preschool children and adults using mice. ACM Transactions on Computer-Human Interaction (TOCHI). 2004;11(4):357–386. doi: 10.1145/1035575.1035577

[21] Jones T. An empirical study of children’s use of computer pointing devices. Journal of Educational Computing Research. 1991;7(1):61–76. doi: 10.2190/2WBH-V235-YA82-VNMC

[22] Crook C. Young children’s skill in using a mouse to control a graphical computer interface. Computers & Education. 1992;19(3):199–207. doi: 10.1016/0360-1315(92)90113-J

[23] King J, Alloway N. Preschooler’s use of microcomputers and input devices. Journal of Educational Computing Research. 1992;8(4):451–468. doi: 10.2190/NBAR-JKPH-DP2G-4M0W

[24] King J, Alloway N. Young children’s use of microcomputer input devices. Computers in the Schools. 1993;9(4):39–54. doi: 10.1300/J025v09n04_05

[25] Joiner R, Messer D, Light P, Littleton K. It is best to point for young children: A comparison of children’s pointing and dragging. Computers in Human Behavior. 1998;14(3):513–529. doi: 10.1016/S0747-5632(98)00021-1

[26] Inkpen KM. Drag-and-drop versus point-and-click mouse interaction styles for children. ACM Transactions on Computer-Human Interaction (TOCHI). 2001;8(1):1–33. doi: 10.1145/371127.371146

[27] Hourcade JP. Learning from preschool children’s pointing sub-movements. In: Proceedings of the 2006 Conference on Interaction Design and Children; 2006. p. 65–72.

[28] Hourcade JP, Crowther M, Hunt L. Does Mouse Size Affect Study and Evaluation Results? A Study Comparing Preschool Children’ s Performance with Small and Regular-Sized Mice. In: Proceedings of the 6th international Conference on interaction Design and Children; 2007. p. 109–116.

[29] Hourcade JP, Perry KB, Sharma A. PointAssist: Helping four year olds point with ease. In: Proceedings of the 7th International Conference on Interaction Design and Children; 2008. p. 202–209.

[30] Strommen EF, Revelle GL, Medoff LM, Razavi S. Slow and steady wins the race? Three-year-old children and pointing device use. Behaviour & Information Technology. 1996;15(1):57–64. doi: 10.1080/014492996120409

[31] Revelle GL, Strommen EF. The effects of practice and input device used on young children’s computer control. Journal of Computing in Childhood Education. 1990;2(1):33–41.

[32] Geist E. Using tablet computers with toddlers and young preschoolers. YC Young children. 2014;69(1):58–63.

[33] Worthen B. What happens when toddlers zone out with an iPad. Wall Street Journal. 2012;.

[34] Ellis K, Power M, Albrecht DW. Toddler techie touch generation. In: Proceedings of the 2018 Annual Symposium on Computer-Human Interaction in Play; 2018. p. 127–139.

[35] Aziz NAA, Batmaz F, Stone R, Chung PWH. Selection of touch gestures for children’s applications. In: 2013 Science and Information Conference. IEEE; 2013. p. 721–726.

[36] Vatavu RD, Cramariuc G, Schipor DM. Touch interaction for children aged 3 to 6 years: Experimental findings and relationship to motor skills. International Journal of Human-Computer Studies. 2015;74:54–76. doi: 10.1016/j.ijhcs.2014.10.007

[37] Nacher V, Jaen J, Navarro E, Catala A, González P. Multi-touch gestures for pre-kindergarten children. International Journal of Human-Computer Studies. 2015;73:37–51. doi: 10.1016/j.ijhcs.2014.08.004

[38] Swinth Y, Anson D, Deitz J. Single-switch computer access for infants and toddlers. The American Journal of Occupational Therapy. 1993;47(11):1031–1038. doi: 10.5014/ajot.47.11.1031 8279498

[39] Uchiyama I, Anderson DI, Campos JJ, Witherington D, Frankel CB, Lejeune L, et al. Locomotor Experience Affects Self and Emotion. Developmental Psychology. 2008;44(5):1225–1231. doi: 10.1037/a0013224 18793056 PMC4067245

[40] Mockler SR, McEwen IR, Jones MA. Retrospective analysis of predictors of proficient power mobility in young children with severe motor impairments. Archives of Physical Medicine and Rehabilitation. 2017;98(10):2034–2041. doi: 10.1016/j.apmr.2017.05.028 28688787

[41] Permobil. Permobil Explorer Mini; 2022. Available from: https://www.permobil.com/en-us/products/power-wheelchairs/permobil-explorer-mini.

[42] Lab MM. Scratch; 2022. Available from: https://scratch.mit.edu/.

[43] LUCI. Modern Mobility; 2020. Available from: https://luci.com.

[44] Michel GF, Campbell JM, Marcinowski EC, Nelson EL, Babik I. Infant hand preference and the development of cognitive abilities. Frontiers in Psychology. 2016;7:410. doi: 10.3389/fpsyg.2016.00410 27047431 PMC4803747

[45] McManus IC. Right hand, left hand: The origins of asymmetry in brains, bodies, atoms, and cultures. Harvard University Press; 2002.

[46] Provost B, Crowe TK, McClain C. Concurrent validity of the Bayley Scales of Infant Development II Motor Scale and the Peabody Developmental Motor Scales in two-year-old children. Physical & Occupational Therapy in Pediatrics. 2000;20(1):5–18. doi: 10.1080/J006v20n01_02 11293915

[47] Jennings KD. Development of goal-directed behaviour and related self-processes in toddlers. International Journal of Behavioral Development. 2004;28(4):319–327. doi: 10.1080/01650250444000036

[48] Plummer T, Logan SW, Morress C. Explorer Mini: Infants’ Initial Experience with a Novel Pediatric Powered Mobility Device. Physical & Occupational Therapy In Pediatrics. 2020;41(2):192–208. doi: 10.1080/01942638.2020.1819935 33019827

[49] Kotsopoulos D, Zambrzycka J, Makosz S. Gender differences in toddlers’ visual-spatial skills. Mathematical Thinking and Learning. 2017;19(3):167–180. doi: 10.1080/10986065.2017.1328634

[50] Schwarzer G, Gehb G, Kelch A, Gerhard-Samunda T, Jovanovic B. Locomotion training contributes to 6-month-old infants’ mental rotation ability. Human Movement Science. 2022;85:102979. doi: 10.1016/j.humov.2022.102979 35952408

[51] Casey BM, Andrews N, Schindler H, Kersh JE, Samper A, Copley J. The development of spatial skills through interventions involving block building activities. Cognition and Instruction. 2008;26(3):269–309. doi: 10.1080/07370000802177177

[52] McLaughlin AC, Rogers WA, Fisk AD. Using direct and indirect input devices: Attention demands and age-related differences. ACM Transactions on Computer-Human Interaction (TOCHI). 2009;16(1):1–15. doi: 10.1145/1502800.1502802 22563232 PMC3342758

[53] Rachwani J, Tamis-LeMonda CS, Lockman JJ, Karasik LB, Adolph KE. Learning the designed actions of everyday objects. Journal of Experimental Psychology: General. 2020;149(1):67. doi: 10.1037/xge0000631 31219298 PMC6923538

